# Wastewater reuse for production of plant-based foods: does spore-forming *Clostridioides difficile* or *Clostridium perfringens* represent a health risk?

**DOI:** 10.1128/aem.00173-26

**Published:** 2026-04-21

**Authors:** Alexandre Mrozinski, Caroline Le Maréchal, Edward Topp

**Affiliations:** 1 Agroécologie laboratory, INRAE, Université Bourgogne Europe 27011, Dijon, France; 2 HQPAP Unit, ANSES, Laboratory of Ploufragan-Plouzané-Niort 355168 , Ploufragan, France; 3 Department of Biology, University of Western Ontario 6221, London, Ontario, Canada; The Pennsylvania State University, University Park, Pennsylvania, USA

**Keywords:** *Clostridioides difficile*, *Clostridium perfringens*, spore, wastewater reuse, crop irrigation, antimicrobial resistance

## Abstract

In the context of drought and increasingly severe water shortages, agriculture faces a major challenge in continuing to feed a growing global population. Irrigation of crops with treated municipal wastewater can contribute to meeting agriculture’s water needs. However, the use of treated wastewater carries numerous public health risks due to the presence of various human and animal pathogens including spore-forming *Clostridium* species, such as *Clostridioides difficile* and *Clostridium perfringens*. Antibiotic resistance in these important pathogens is an emerging issue. Spores of these bacteria are extremely persistent, and they can persist for years in a wide range of environments. Their presence in treated wastewater could lead to contamination of soil and of plant-based foods grown for human or animal consumption. Within this context, the present review addresses the pathogenicity of *C. difficile* and *C. perfringens* and their potential role in the dissemination of antimicrobial resistance in the context of wastewater reuse for agriculture.

## INTRODUCTION

Water is an essential resource for both human activities and ecosystems, yet its availability is increasingly threatened by climate change. As global temperatures rise, prolonged droughts and irregular precipitation patterns are becoming more frequent leading to significant water shortages in many regions. This poses a significant challenge for water-intensive agriculture, especially during spring and summer when water scarcity is most acute. Irrigation of crops with treated municipal wastewater (TWW) is an agricultural practice that will help alleviate water shortages that now threaten food security (1) by ensuring a constant and reliable water supply. Recent regulatory policy changes undertaken in Europe are designed to encourage this practice, which is already widely used in some other countries such as Australia and Israel (2).

Clearly, given that wastewater treatment plants (WWTPs) process fecal material, the microbiological water quality of the effluent is a critical factor of public health concern when TWW is used to irrigate crops intended for animal and, in particular, human consumption. Microbiological water quality standards at the national or European Union levels specify the maximum limits of some microorganisms that are permissible for irrigating crops to be consumed by humans (2). It is assumed that consuming plant-based foods irrigated with water of this quality poses no, or little, risks of enteric illness. Unfortunately, in much of the world, there is currently little or no wastewater treatment infrastructure. In 2020, 44% of the household wastewater generated globally was discharged without safe treatment (3) and 10% of the global population are estimated to consume plant-based foods irrigated with wastewater (4). Thus, wastewater effluent and environments receiving effluent are heavily contaminated with enteric microorganisms (5–7). Under these circumstances, it is impossible to meet the TWW Class A quality standard mandated by the European Union, and the risk of microbiological contamination of crops with enteric microorganisms in these settings is clearly significant. Molecular and cultural approaches reveal the presence of multiple pathogenic bacteria, including *Shigella*, *Legionella*, *Leptospira*, *Campylobacter*, *Yersinia*, and *Clostridium* spp., in effluent (8, 9). The Clostridia are notable in three respects. They are obligate anaerobes, spore-forming, Gram-positive, toxin producers with numerous species that are important pathogens, including *Clostridium tetani* (causative agent for tetanus), *Clostridium botulinum* (botulism), *Clostridium perfringens* (gastroenteritis), and *Clostridioides difficile* (formerly *Clostridium difficile* [10]) (acute gastrointestinal illness). In this review, we focus on pathogens that can be transmitted to humans through food consumption of products irrigated with TWW, specifically *C. difficile* and *C. perfringen*s. Other *Clostridium* species are not considered, as the diseases they cause are not associated with the ingestion of bacteria via contaminated food. For example, *C. tetani* is not a foodborne pathogen; it is primarily associated with bite injuries and traumatic or surgical wound infections, where toxin production occurs locally at the site of infection (11, 12). Similarly, *C. botulinum* and, more broadly, botulinum neurotoxin-producing Clostridia are excluded from this review. Foodborne botulism results from the ingestion of preformed toxins present in contaminated food, produced by neurotoxin-producing bacteria particularly *C. botulinum* rather than from ingestion of the bacteria themselves (13).

Although these species differ in their routes of transmission, they share a key ecological trait: the production of highly resistant spores capable of long-term environmental persistence, including during wastewater and biosolid treatment (14–16). Moreover, *Clostridium* spp. can persist in soils for at least a decade following manure application (17). The window of risk for crop exposure to these spores will, therefore, be much wider than that for non-spore-forming pathogens that die off much more rapidly in soil (18).

Given the increasing threat to food security from climate change, the ubiquity of *C. difficile* and *C. perfringens* in sewage, the remarkable persistence of their spores during sewage treatment and in soil, this review considers the potential contamination with these bacteria of crop production systems irrigated with TWW. The underlying concern is that these pathogens will be transmitted to humans through the consumption of TWW-irrigated plant-based foods.

## PUBLIC HEALTH CONCERNS AND PATHOGENIC POTENTIAL

### Clostridioides difficile

*Clostridioides difficile* infection (CDI) is a disease primarily involving the large intestine (19). It typically occurs following treatment with antibiotics that alter the composition of the gut microbial community (dysbiosis), reducing the resilience to infection, thereby permitting *C. difficile* to proliferate. Approximately 5% of adults and 15%–70% of children are colonized by *C. difficile* including asymptomatic carriers (20). The most common symptom of CDI is acute and chronic diarrhea, with clinical conditions ranging from mild colitis to fulminant colitis and even death (21, 22). A CDI causes substantial excess mortality, with about 74% of older patients (≥65 years) and 25% of younger patients (<65 years) dying in Sweden, but the relative excess risk is far greater in younger individuals than in older ones (23). More than 35% of patients who receive treatment for an initial CDI episode are expected to experience a recurrence (rCDI), and approximately 65% of those with a first recurrence are likely to face further episodes (24). *C. difficile* is classified by the U.S. Centers for Disease Prevention and Control (CDC) as an urgent threat, with more than 223,000 cases of CDI and 12,800 deaths in the United States in 2017 (25). The primary transmission of *C. difficile* occurs via the fecal oral route and is facilitated by its sporulation mechanism and by the environmental recalcitrance of spores (20).

In Europe, the incidence of hospital-acquired *C. difficile* infection (HA-CDI) is increasing, with 2.02 cases per 10,000 patient-days in 2018 and 2.58 in 2020 (26). While HA-CDI has been decreasing in recent years in the United States, the global prevalence of CDI remains stable. In contrast community-acquired *C. difficile* infection (CA-CDI), defined as the onset of diarrhea in the community or within 48 hours of hospitalization, without a history of discharge from a healthcare facility in the preceding 12 weeks is currently on the rise. According to the CDC, the estimated incidence of CA-CDI in the United States increased from 52.88 per 100,000 people in 2012 to 62.1 per 100,000 people in 2022 (92.90 in 2012 and 54 in 2022 for HA-CDI) (27, 28). The same trend is observed in Europe for CA-CDI (or unknown association) with an incidence of 0.69 per 1,000 patients in 2019 and 1.35 in 2020, with Germany having higher incidence than other European countries (26). Epidemiological studies indicate that patients diagnosed with CA-CDI tend to be younger, with a median age of 50 years, compared to 72 years for HA-CDI. Moreover, CA-CDI appears to predominantly affect females, with 76% of cases occurring in women, compared to 60% in HA-CDI. In terms of clinical outcomes, CA-CDI is associated with lower mortality rates than HA-CDI. An important finding is that 36% of CA-CDI patients report no antibiotic exposure in the 12 weeks prior to diagnosis, suggesting additional potential risk factors contributing to disease development (29, 30). Moreover, this number is likely an underestimate because of the absence of diagnosis following long-term diarrhea.

The main virulence factors for *C. difficile* are two potent toxins, toxin A (enterotoxin; TcdA) and toxin B (cytotoxin; TcdB). Hypervirulent strains of *C. difficile* frequently produce a third toxin called *C. difficile* binary toxin (CDT with two locus: CdtA and CdtB) (31). These toxins are encoded by genes generally located on the chromosome, carried by two distinct loci: PaLoc and CdtLoc (32). Recent findings have also revealed the presence of *C. difficile* strains carrying a plasmid that contains both *tcdB* and the CdtLoc (33, 34), and a temperate phage harboring the CdtLoc has been characterized (35). Interestingly, it has been shown that the PaLoc can be transferred between *C. difficile* strains (36). This suggests the existence of horizontal transfer of toxin-encoding genes and the dissemination of virulence factors among *C. difficile* strains.

Worryingly, there is recent evidence suggesting that *C. difficile* may be associated with an increased risk of colorectal cancer. Its toxins, in particular TcdB, may, indeed, play a key role in the development of the cancer (37, 38). As colorectal cancer is the second leading cause of cancer-related deaths in the United States, with its incidence rising each year among young adults (39), these findings underscore the need for further research into the potential implications of CDI in colorectal cancer development.

*C. difficile* has historically been classified using pulsed-field gel electrophoresis (PFGE) and PCR ribotyping, with more than 600 ribotypes (RTs) currently identified (40). This latter method is still considered the gold standard for *C. difficile* characterization. This technique is based on the amplification of the intergenic spacer region between the 16S and 23S rRNA genes, followed by analysis of the resulting banding patterns to differentiate strains (41). A shared database WEBRIBO allows comparison and identification of ribotypes worldwide although no standardized laboratory protocol currently exists for performing the assay. More recently, multilocus sequence typing (MLST) has been employed for *C. difficile* classification. This approach has enabled the delineation of *C. difficile* into ten monophyletic clades: five main clades (C1, C2, C3, C4, and C5) and five so called cryptic clades (C-I, C-II, C-III, C-IV, and C-V) (42, 43). Clades 1 to 5 appear to be primarily associated with human and animal disease, whereas the cryptic clades are more commonly linked to environmental isolates (33, 34, 42–47). *C. difficile* strains from cryptic clades could represent populations adapted to environmental reservoirs, with limited pathogenic potential in humans or animals (42, 43, 47, 48). Nevertheless, the prevalence of these cryptic clades may be underestimated, primarily due to their atypical toxin gene architectures, which can lead to the failure of current diagnostic assays (33, 42, 43). Cryptic clades may harbor a PaLoc monotoxin variant lacking *tcdA*, in combination with a CdtLoc region (42, 43), but the ability of strains producing this variant TcdA to induce clinical signs in humans has not been addressed yet although this might be expected as it induces weakly positive results in the commercial immunoassays. Current databases contain only 0.2% of genomes from cryptic clades, compared to 99.8% from classical clades, including 57.2% belonging to clade C1 (42). Average Nucleotide Identity (ANI) provides an accurate method for species delineation, defining monophyletic groups whose genomes share at least 95% similarity (49). This method has recently been applied to *C. difficile* clades, showing that clades C1–C4 have an ANI >97%, while clade C5 lies at the borderline of the accepted species threshold (95.9%–96.2%) (42). More interestingly, the five cryptic clades display ANI values clearly below 96%, with ANI values between C-I, C-II, and C-III themselves being <96%, indicating that they are as divergent from each other as from *C. difficile* (42, 43). Comparative ANI analysis of the cryptic clades against more than 5,000 reference genomes across 21 phyla failed to identify a closer match than *C. difficile* itself (89%–94% ANI) (42). These results indicate that the classification of *C. difficile* remains under active investigation, particularly for environmental strains that may warrant reclassification under different species names (42, 43). *C. difficile* environmental strains appear to differ markedly from those associated with human clinical cases.

### Clostridium perfringens

*Clostridium perfringens*, one of the most widespread bacterial species with a ubiquitous environmental distribution, is remarkable for its ability to produce at least 20 different toxins in various combinations (50, 51). Each toxinotype of *C. perfringens* is characterized by a primary disease associated with humans and/or animals, as briefly summarized in Table 1. *C. perfringens* is estimated to be responsible for roughly one million cases of gastroenteritis annually, ranking it as the third most prevalent bacterial cause of foodborne illness in the United States, with close to two million cases per year (52). Additionally, *C. perfringens* is the primary cause of traumatic gas gangrene, primarily associated with war injuries and surgical wounds (52, 53). *C. perfringens* strains are classified into seven toxinotypes (from A to G) based on their capacity to produce toxins (Table 1). There are six principal toxins of *C. perfringens*: α (CPA), β (CPB), ε (ETX), ι (ITX), CPE, and NetB (Table 1). All strains produced the α toxin, the most important virulence factor involved in human gas gangrene. The β, ε, ι, and NetB toxins are encoded by genes located on plasmids, whereas the α toxin gene, present in all *C. perfringens* strains, is chromosomally encoded. The *cpe* gene can be located either on the chromosome or on a plasmid (54), and *C. perfringens* strains with a chromosomal *cpe* gene are most commonly associated with foodborne intoxications, whereas those carrying a plasmid-borne *cpe* gene are more frequently linked to non-foodborne gastrointestinal infections. It should be noted that *C. perfringens* strains with a chromosomal *cpe*, typically implicated in foodborne outbreaks, are significantly more resistant to inactivation treatments (such as heat and high pressure) and preservation methods (such as salt and nitrites) than enterotoxigenic strains harboring a plasmid-borne *cpe* gene (55).

**TABLE 1:** Summary of *C. perfringens* toxinotypes: genetic carriage and associated diseases in various hosts (53, 54, 56, 57)

Type	Toxins						Diseases in
	α (CPA)	β (CPB)	ε (ETX)	ι (ITX)	CPE	NetB	
A	+						Humans, sheep, cattle, horses
B	+	+	+				Sheep, cattle, horses
C	+	+			+/−		Humans, cows, pigs, lambs, calves, foals, etc.
D	+		+		+/−		Sheep, goats, cattle
E	+			+	+/−		Rabbits, lambs, cattle
F	+				+		Humans, horses, dogs, pigs, foals, goats
G	+					**+**	Chickens, turkeys
Genetic support	Chromosomal	Plasmid	Plasmid	Plasmid	Chromosomal/plasmid	Plasmid	
Biological activities	Necrotizing, hemolytic, contraction of smooth muscle	Dermonecrosis, edema, enterotoxic	Dermonecrosis, edema, contraction of smooth muscle	Necrotizing	Erythema, enterotoxic	Hemolytic	
Associated diseases	- Gas gangrene - Sudden infant death syndrome - Necrotic enteritis (chickens) - Enteritis (calves, piglets) - Inflammatory diseases	- Necrotizing enteritis (human, pigs, sheep, goats, and calves)	- Enterotoxaemia (sheep, goats)	- Lethal necrotizing - Enteritis and sudden death in beef calves	- Foodborne illness (humans) - Enteritis necroticans (humans) - Antibiotic-associated diarrhea - Sporadic diarrhea - Enteric diseases (swines, cattles, horses, sheep, goats, deers, bears)	- Necrotic enteritis (chickens)	

Many of these toxins are encoded by the family of conjugative plasmids pCW3/pCW3-like, and a single strain can simultaneously carry multiple plasmids, each harboring different toxin genes (53, 58). Plasmid transfer has been demonstrated *in vitro* between *C. perfringens* allowing the acquisition of toxin genes (59, 60). These plasmid transfer phenomena could lead to the emergence of new toxigenic populations in both humans and animals, through the consumption of a pathogenic strain carrying a plasmid that itself contains a toxin gene that can be carried to the resident *C. perfringens* strains in the intestines (53, 59).

To the best of our knowledge, no study focusing on the characteristics of environmental *C. perfringens* strains or on their specificities has been published yet.

### Antimicrobial resistance of *Clostridioides difficile* and *Clostridium perfringens*

There are currently only two antibiotics available to treat human CDI: vancomycin and fidaxomicin (61). Metronidazole was previously the first-line treatment for CDI, but it is no longer recommended by the European Society of Clinical Microbiology and Infectious Diseases (ESCMID) due to its association with infection recurrence, neurotoxicity, and increasing resistance notably with the propagation of the plasmid pCD-METRO conferring resistance to this antibiotic (61–64). As a last resort treatment for rCDI, microbiota-based therapies such as fecal microbiota transplantation (FMT) can be used and have been shown to be effective. FMT helps restore the diversity of the intestinal microbiota and has a success rate of around 90% in treating rCDI in patients who have not responded to multiple rounds of antibiotic therapy (65). In terms of antibiotic resistance from a therapeutic perspective, *C. perfringens* is less problematic than *C. difficile* for two reasons: *C. perfringens* infections (CPI) usually resolve spontaneously, and the use of antibiotics for the treatment of CPI is not recommended except in more severe cases such as gangrene or sepsis (66).

Although resistance to vancomycin and fidaxomicin is still considered uncommon in *C. difficile*, there are emerging resistant isolates involving the *vanG* operon in the case of vancomycin (67) and likely mutations within the *rpoB* gene for fidaxomicin (68). Apart from the clinical aspect, *C. difficile* has resistance to other antibiotics not used to treat CDI. These include the fluoroquinolones (mutations on *gyrA*, *gyrB*), clindamycin (*ermB* which can be carried by a transposon), tetracycline (numerous efflux pumps and/or *tetA(P*), *tetB(P)*, *tetM*, t*etW* which can be carried by a transposon), carbapenems (mutations on *pbp1*, *pbp3*), macrolide‐lincosamide‐streptogramin B (MLS) (*erm* genes, *cfr* genes, *msrA*, and others that can be carried by transposons, or plasmids), and numerous β‐Lactams (penicillins, monobactams, all cephalosporins) with the presence of chromosomal class D β-lactamase genes (blaCDDs) (61, 69).

Antibiotic resistance is commonly observed as well in *C. perfringens*, and many of these resistant strains carry plasmids (e.g., pCW3/pCW3-like plasmids). These plasmids frequently harbor genes conferring resistance to several classes of antibiotics, including tetracyclines (*tetA(P)* and *tetB(P)*), chloramphenicol (*catP*), macrolide–lincosamide–streptogramin antibiotics (*erm(B)* and *erm(Q)*), as well as specific resistance to lincomycin (*lnuP*). Resistance to bacitracin has also been associated with the *bcrRABD* locus (58).

Many resistance genes in *C. difficile* and *C. perfringens* are associated with MGEs. For example, in a study of 2,190 genomes of *C. difficile* from the GenBank database, 1,101 (50%) carried one or more mobile genetic elements (MGEs), and 48% (488/1,101) of these carried antimicrobial resistance (AMR) genes (70). *C. perfringens* can carry many plasmids related to pCW3, which carry genes conferring resistance to chloramphenicol, erythromycin, lincomycin, bacitracin, or clindamycin (58). This high prevalence of MGE could facilitate the dissemination of antimicrobial resistance both within species and potentially to other clinically relevant pathogenic bacteria (58, 61, 63).

Several studies have pointed out that Clostridia may play a role in the dissemination of AMR genes in soil regularly amended with manure (17, 71, 72). Moreover, *C. perfringens* has been suspected of being a reservoir for conjugative elements carrying AMR genes (73–75) and *C. difficile* a reservoir of AMR genes that might be transferred to other species in the human gut (76, 77). Despite this, the AMR reservoir within Clostridia remains poorly characterized, particularly regarding the potential for horizontal gene transfer and its implications for treatment efficacy (78, 79). Furthermore, the evaluation of these bacterial species within the framework of antimicrobial risk assessment remains an underexplored area. Should this risk be considered negligible, or does it represent a significant knowledge gap that warrants further investigation?

## ENVIRONMENTAL PERSISTENCE AND DISSEMINATION OF *CLOSTRIDIOIDES DIFFICILE* AND *CLOSTRIDIUM PERFRINGENS*

Numerous studies have detected Clostridia in diverse soil types across the globe. These bacteria are recognized as major soil-borne pathogens capable of causing severe, and sometimes fatal, infections. Notably, their spores can persist in the environment for many years, contributing to their widespread distribution and resilience (80). Due to their capacity to form spores, *C. difficile* and *C. perfringens* are extremely recalcitrant and persistent. Moreover, Clostridia can survive heat treatments like pasteurization and thermophilic digestion, processes meant to reduce or eliminate pathogens (80)*, C. perfringens* spores can survive in boiling water for an hour or even longer (81). This poses a significant challenge to the food industry, as it increases the risk of contamination and foodborne outbreaks despite cooking and chilling. Classified as an aerotolerant anaerobe, *C. perfringens* can survive in aerobic environments and can initiate disease development under aerobic conditions (82). This bacterium grows very rapidly during infection with a doubling time of 12–17 min, giving it a decided competitive advantage against other resident bacteria, resulting in effective gut colonization (82, 83).

The persistence of *C. difficile* and *C. perfringens* in food and environmental sources underscores the need to investigate other potential reservoirs, particularly wastewater, which may play a key role in their transmission dynamics. *C. difficile* was detected in the effluent from nine WWTPs in Switzerland (84). Twenty-seven isolates were characterized, 92% of which were toxigenic, carrying *tcdA*, *tcdB*, or *cdtAB* (84). A survey of 20 WWTPs effluents in England revealed 18 to be positive for *C. difficile*. Fifty-six percent of 186 isolates were toxigenic (85). Forty-eight percent of 104 effluent samples from 12 WWTPs surveyed in Australia were positive for *C. difficile*, 30% of which were toxigenic (16). *C. perfringens* is considered an indicator of TWW quality and is used as a proxy for protozoan contamination in France and across Europe. In a survey of effluent from three WWTPs in Hamburg, Germany, *C. perfringens* was detected in 10 of 20 effluent samples (14). A Japanese survey found *C. perfringens* strains carrying the *cpe* gene in 56% of 36 effluent samples (86). Thirty- seven strains were obtained, 57% of which tested positive for the beta2 toxin gene (*cpb2*).

**Fig 1:**
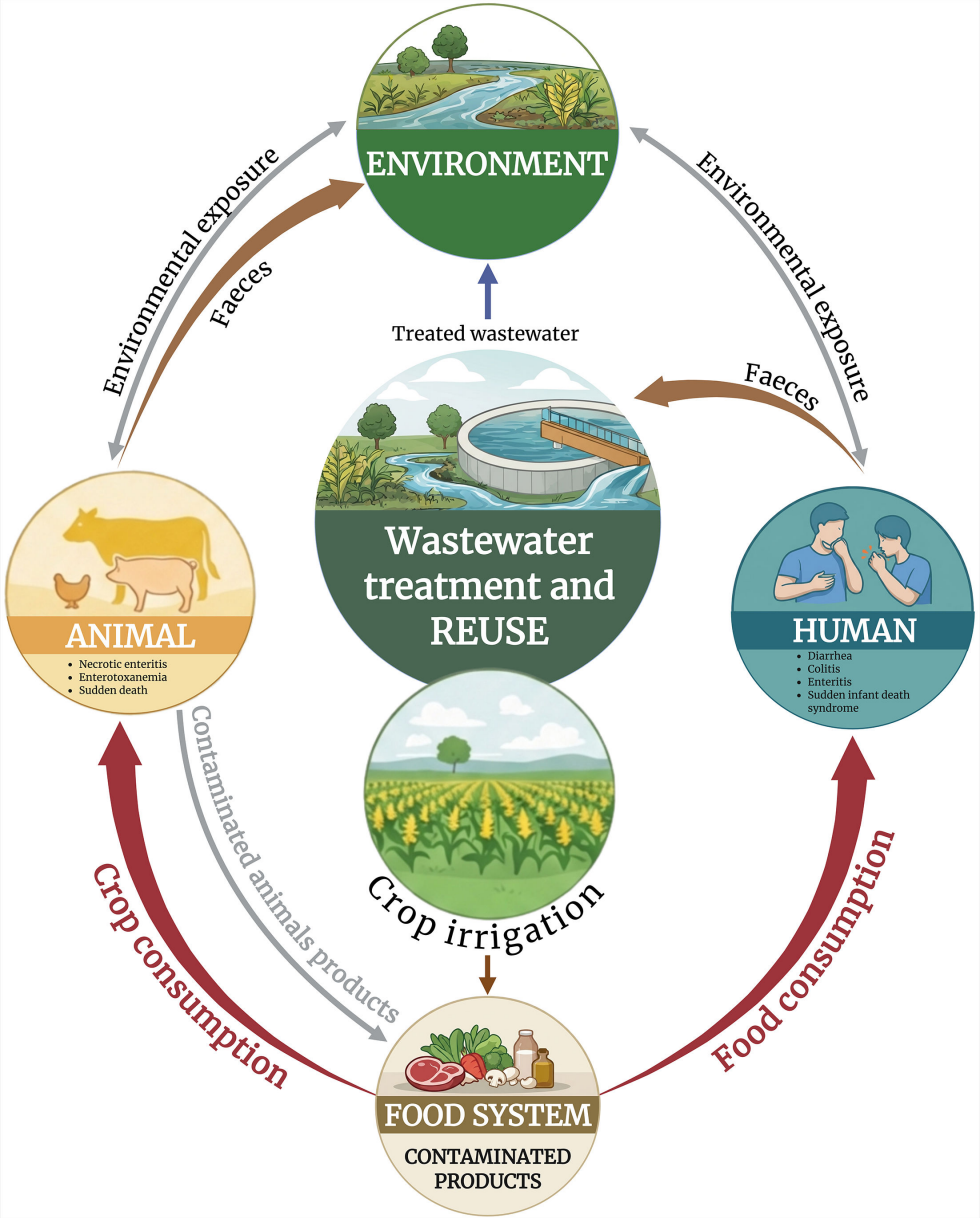
Event diagram summarizing potential routes of transfer of *Clostridioides difficile* and *Clostridium perfringens* from wastewater to consumers via foodstuffs. Created in Biorender. Biorender, H. (2026) https://BioRender.com/ou1jc0x.

Despite the low number of available studies (Table 2), these findings clearly indicate the presence of both *C. difficile* and *C. perfringens*, including toxigenic isolates in WWTP effluents. The reuse of TWW for crop irrigation could therefore pose a health risk, particularly if these spore-forming bacteria persist during cultivation. The available data about the detection of *C. difficile* and *C. perfringens* in TWW raise the question of their dissemination in agricultural systems through TWW reuse and more broadly in the environment, notably in soil, food production (leading to human and animal exposure, as well as rivers and the sea through water runoff [Fig. 1]). An additional dissemination pathway is the airborne spread of bacteria during sprinkler irrigation, particularly in crops such as corn. This mode of spread could not only affect the irrigated field but also surrounding areas, including home gardens nearby, potentially posing both environmental and health risks.

**TABLE 2:** Summary of studies on *Clostridioides difficile* and *Clostridium perfringens* in WWTP (wastewater treatment plant) effluents

Species	Presence in effluent	Toxin information	Citation
*C. difficile*	48.1% (50/104) (12 WWTPs, 104 effluent samples)	30.8% (32/104 isolates) of isolates from effluents were toxigenic. Including influent, effluent, and biosolids (284 isolates): 52.8% (150/284 isolates) *tcdA*+ /t*cdB*+ of which 8.7% (13/150 isolates) were also *cdtA*+ /*cdtB*+. 44.7% (127/284 isolates) were non-toxigenic (*tcdA*− /*tcdB*− /*cdtA*− / *cdtB*−). Seven isolates had particular toxinotype: *tcdA*− / *tcdB*− / *cdtA*+ / *cdtB*+ (1.4% [4/284 isolates]) and *tcdA*− / *tcdB*+ / *cdtA*+ / *cdtB*+ (1.1% [3/284 isolates])	(16)
*C. difficile*	100% (9 WWTPs, 1 effluent sample/WWTPs)	43.6% (24/55 isolates) *tcdA*+ / *tcdB*+ / *cdtA*− / *cdtB*− 41.8% (23/55 isolates) *tcdA*+ / *tcdB*+ / *cdtA*+ / *cdtB*+	(84)
*C. difficile*	100% (1 WWTP, 12 effluent samples/WWTPs)	37.2% (76/121 isolates) toxigenic (no precision) 3.3% (4/121 isolates) had *cdtA* and *cdtB*	(87)
*C. difficile*	100% (1 WWTP, 5 effluent samples)	100% *tcdB*+ (no more precision)	(88)
*C. difficile*	100% (18 WWTPs, 1 effluent sample/WWTPs)	56% (106/186 isolates) *tcdA*+ / *tcdB*+ / *cdtA*+ / *cdtB*+ 32% (60/186 isolates) 56% (106/186) *tcdA*− / *tcdB*− / *cdtA*− / *cdtB*− 8% (8/186 isolates) *tcdA*+ / *tcdB*+ / *cdtA*− / *cdtB*− 2% (5/186 isolates) 56% (106/186) *tcdA*− / *tcdB*− / *cdtA*+ / *cdtB*+ 1% (2/186 isolates) 56% (106/186) *tcdA*− / *tcdB*+ / *cdtA*− / *cdtB*−	(85)
*C. perfringens*	40% (1 WWTP, 10 effluent samples)	ND^*a*^	(14)
*C. perfringens*	56% (2 WWTPs, 36 effluent samples)	56% (20/36 isolates) *cpe*+ 57% (21/37 isolates) *cpb2*+	(86)
*C. perfringens*	100%	ND	(89)

^*a*^ ND, no data.

Persistence in soils of *C. difficile* and *C. perfringens* after manure application has been reported. Using WGS, it has been shown that *C. difficile* from poultry manure was still detected in the soil 2 years after spreading operations (90). Such persistence has also been reported for Clostridia in soils exposed to swine manure (17). Although it has not been investigated at this time, reuse of TWW could have a similar impact by carrying Clostridia and facilitating their dissemination into agricultural fields. The environmental factors that promote the survival and growth of *Clostridia* spp. in soil have been reviewed (80). In summary, some Clostridia species can grow across a wide range of temperatures, with the most pathogenic species thriving at an optimum range of 30–40°C. Certain strains can grow at temperatures as low as 10°C or below. For instance, *C. perfringens* has been detected in Antarctic soils, where temperatures range from −38°C to 3.2°C (91). Warmer temperatures increase metabolic activity in soil, raising oxygen demand and potentially creating anoxic conditions, particularly in wet soils, which favor the growth of anaerobic bacteria (92) such as Clostridia, allowing them to multiply and produce toxins. In deleterious situations such as aerobic conditions, sporulation will allow the survival of the bacteria and, therefore, its persistence in the environment.

The contamination of crops intended for human consumption by *C. difficile* and/or *C. perfringens*, whether through vegetative cells or spores, poses a significant public health risk. Consuming contaminated crops could lead to foodborne illnesses. Crops can also be used as feed for animals, leading to animal contamination and potential transmission to humans through the food chain (Fig. 1). This, in turn, may contribute to the spread of Clostridia through human and animal wastes, creating a cycle of contamination as the pathogens re-enter the soil and water systems. Crops irrigated with TWW can be contaminated with *C. difficile* and/or *C. perfringens* through soil present on the product. A study of the surface attached soil of commercially available potatoes in Japan revealed that *C. perfringens* spores were present on 83% of the samples (93). They obtained 288 isolates of which 64 (22%) were positive for *cpe*. Potatoes sourced from 12 European countries were also examined, and 22.4% of the samples tested positive for *C. difficile* (94). They reported a significant difference in the proportion of *C. difficile* positive samples between potatoes with soil attached and those without. An Australian survey of retail vegetables revealed that approximately 50% of potatoes and 5% of onions and carrots, crops typically grown in soil, were contaminated with *C. difficile*. Around half of the isolates were toxigenic, and several ribotypes overlapped with those identified in human and animal sources (95). Surface-attached soil acts as a dissemination vector for Clostridia within consumers' homes. Although raw potatoes with soil attached are not typically eaten, the soil can be dispersed within the kitchen during cleaning or cutting. This dispersion can lead to the contamination of kitchen surfaces, such as countertops and cutting boards, which, in turn, may contaminate other types of food, increasing the risk of bacterial transmission. Cross-contamination of foodstuffs via cutting boards used to process raw poultry products carrying *Campylobacter* spp. is documented (96). A similar mechanism has been hypothesized for *C. difficile* (97). Although internalization of pathogenic bacteria such as *Salmonella* spp. in plant tissues of leafy greens occurs (98) and clover roots can be colonized by *C. botulinum* (99), the possible internalization of *C. difficile* or *C. perfringens* has not, to our knowledge, been investigated. Internalized bacteria in vegetables eaten raw will not be removed by standard culinary practice and will be ingested by the consumer (100).

Through these various pathways of Clostridia dissemination in the food chain, the major risk lies with ready-to-eat vegetables and fruits that are consumed raw and, thus, bypass the cooking process that would otherwise kill the bacteria. But depending on the cooking method, some spores that resist high temperatures can make certain prepared dishes a vector of contamination.

As indicated above, *C. difficile* and *C. perfringens* can carry many MGEs with toxins or AMR genes. With a higher prevalence in the food chain in addition to causing infections, these bacteria could acquire or transfer toxins or AMR genes to other resistant bacteria in soil or in the gut. The previous demonstration that resident gut *C. perfringens* can acquire virulence genes from toxigenic strains in the food chain (59, 60) highlights a potential risk for the emergence of more virulent resident strains and severe infections.

## MITIGATION STRATEGIES AND REGULATORY FRAMEWORK

The European regulation 2020/741 on water reuse for agricultural irrigation establishes four classes of reclaimed water quality (A to D), each corresponding to specific crop types and irrigation methods. Class A applies to all food crops eaten raw, including root vegetables, and requires the highest water quality (*E. coli* ≤ 10 CFU/100 mL, BOD₅ [biochemical oxygen demand on 5 days] ≤ 10 mg/L, TSS [total suspended solids] ≤ 10 mg/L, turbidity ≤ 5 NTU [nephelometric turbidity unit]). Class B covers crops eaten raw where the edible part is not in contact with the water, as well as processed and non-food crops, with *E. coli* ≤ 100 CFU/100 mL. Class C applies to similar crops as Class B but only when using irrigation methods that avoid contact with edible parts (*E. coli* ≤ 1,000 CFU/100 mL). Class D concerns industrial, energy, and seed crops, allowing up to *E. coli* ≤ 10,000 CFU/100 mL. Across all classes (A to D), reclaimed water must also meet the requirement of <1,000 CFU/L of *Legionella* spp. when aerosolization may occur and ≤1 helminth egg/L when used for irrigating pastures or forage crops.

Before reclaimed water can be used, European regulations require that the treatment process must be validated to demonstrate its microbiological performance. This validation requires achieving at least a 5 log₁₀ reduction in *E. coli* (indicator for pathogenic bacteria), a 6 log₁₀ reduction in coliphages (indicator for viruses), and a 4 log₁₀ reduction in *C. perfringens* spores (indicator for protozoan pathogens). These log₁₀ reduction values are calculated relative to the concentration of microorganisms in the raw influent water entering the treatment plant, showing how effectively the process reduces the microbial load between the untreated wastewater (influent) and the reclaimed water (effluent) ready for reuse. These validation tests are performed during the initial qualification of the treatment system or when it is modified, not during routine monitoring. Once validated, regular monitoring ensures that the reclaimed water continues to meet the quality standards defined for its class. This monitoring includes frequent analyses of microbiological and physical parameters, with stricter requirements for higher-quality classes. Together, these measures ensure that reclaimed water is safely matched to its agricultural use, protecting both public health and the environment.

Although the enumeration of *C. perfringens* is included in current regulations, there are, to date, no requirements to assess the toxigenic potential of *C. perfringens* strains or of *C. difficile* in TWW. Methods recommended by the regulation provide information on bacterial abundance, but not on their virulence or associated health risk.

To evaluate the persistence of anaerobic spores, a widely used method is the enumeration of sulfite-reducing Clostridia (SRC), which is a reliable and cost-effective approach. It relies on the ability of many *Clostridium* spp. to reduce sulfite to sulfide (101). However, current evidence indicates that SRC cannot be considered a quantitative surrogate for *C. perfringens*, and even less so for *C. difficile*, in TWW. The SRC group is taxonomically heterogeneous and is frequently dominated by species originating from soil and other environmental reservoirs (e.g., *C. bifermentans, C. sporogenes*, *C. butyricum*). This heterogeneity reflects the broad distribution of the sulfite-reducing phenotype across the diverse *Clostridium* lineage, including numerous species formerly assigned to the genus *Clostridium* that have since been reclassified into new genera (101). Consequently, SRC enumeration may serve as an indicator of treatment performance for spore removal and barrier robustness, but it cannot be used as a direct indicator of the health risks associated with *C. perfringens* or *C. difficile*.

When pathogen-specific information is required for risk assessment, targeted monitoring of *C. perfringens* using standardized culture-based methods is technically feasible. In contrast, routine monitoring of *C. difficile* remains impractical due to the lack of universal standardized methods, complex anaerobic culture requirements, and the uncertain relationship between environmental detection and human health risk. It should also be noted that enumeration and confirmation of these two pathogens involve significant analytical costs and laboratory expertise. Molecular techniques may be applied as complementary tools, targeting genes such as *plc* (*cpa*) for species-level detection of *C. perfringens*, *cpe* for toxigenic strains, *gluD* or *tpi* for species-level detection of *C. difficile*, and *tcdA*/*tcdB* for toxigenic strains. However, DNA-based detection provides information on total DNA present in the sample and does not demonstrate viability or infectivity; therefore, results must be interpreted with caution, ideally in combination with culture-based methods or viability PCR approaches.

Overall, there is a clear need for further studies to develop scientifically robust, risk-based indicators that focus on identifying viable and toxigenic strains of *C. difficile* and *C. perfringens* in TWW, rather than merely enumerating total bacterial counts. Such research is essential to evaluate whether current surrogate markers are sufficient or whether pathogen-specific detection of hazardous strains should be incorporated into future regulatory frameworks.

**Fig 2:**
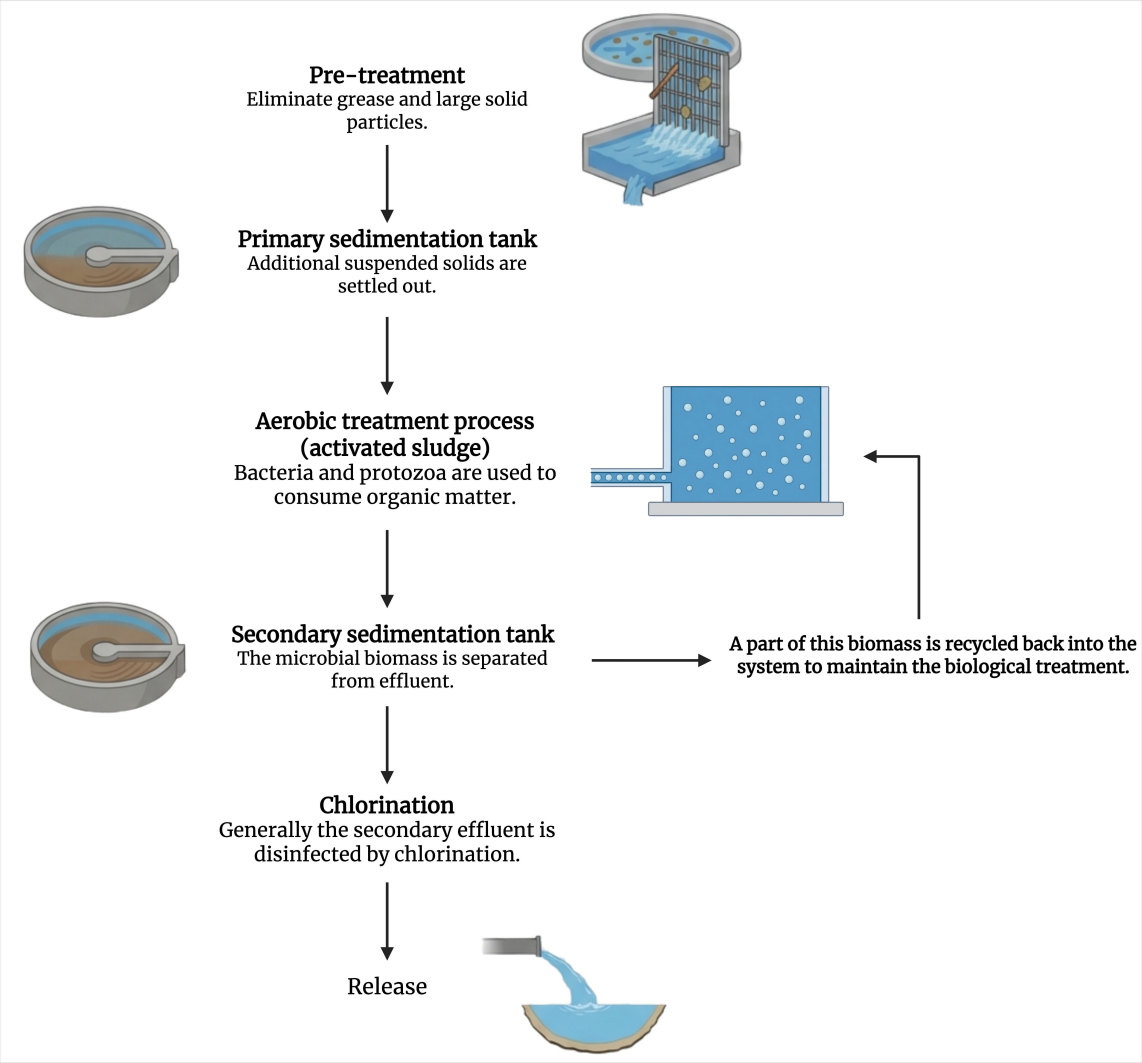
Schematic overview of conventional wastewater treatment processes (102). Created in BioRender. Biorender, H. (2026) https://BioRender.com/8nssutg.

WWTPs follow several key steps (102): pre-treatment removes large solids and grease, followed by primary sedimentation. Aerobic treatment with activated sludge breaks down organic matter (103). Secondary sedimentation separates biomass, part of which is recycled. Some systems also remove nitrogen through nitrification/denitrification. Final disinfection, typically via chlorination, precedes discharge into natural water bodies (Fig. 2). In addition to the activated sludge process, WWTPs can implement additional tertiary/quaternary treatments. Chlorination is a widely used disinfection method in the final stages of sewage treatment, effectively reducing pathogens like *E. coli* by 0.7 to 2.6 log (104). *C. difficile* can survive through the process of WWTPs using chlorination (105), and *C. perfringens* is known to be resistant to this chemical (106). High-intensity ultraviolet (UV) light can be used to eliminate bacteria, protozoa, viruses, molds, yeasts, fungi, nematode eggs, and algae (107). Combined with other processes, UV has been widely studied as a tertiary treatment method and demonstrated that UV light kills bacteria in proportion to its intensity (108). Ozonation processes are used to react with organic contaminants/micro-pollutant and degrade them (102, 109). This treatment was able to reduce the CFU/mL of *E. coli*, *Enterococci*, *Acinetobacter baumannii,* and ESBL compared to conventional treatment, but looking at the 16S rRNA gene marker, ozonation was less efficient compared to ultrafiltration (explained below) (109). We found no data regarding these last two treatments for Clostridia. Membrane filtration methods include a diverse group of processes, usually using pressure-driven membranes through semi-permeable membranes. Four main types of membrane filtration are commonly distinguished: microfiltration (with pore sizes around 1 µm), ultrafiltration (20 nm), nanofiltration (1 nm), and reverse osmosis (which relies on a semi-permeable membrane with pores small enough to remove even dissolved salts and molecules) (102, 109).

The persistence of bacteria following different membrane filtration processes has been studied and has shown that membrane filtration methods are capable of reducing *E. coli* from wastewater (110). The percentage of bacterial removal is suggested to increase as the membrane pore size decreases, with nanofiltration and reverse osmosis being the most effective (111). Integrating membrane systems may be a very efficient system to treat WW and eliminate bacteria. Effluents from WWTPs employing both microfiltration and reverse osmosis were found to have no detectable *C. difficile* (16). Nevertheless, 10% of effluents from WWTPs using membrane filtration (in addition to chlorination and ultraviolet) without reverse osmosis were tested positive for *C. difficile*. It was observed that ultrafiltration was unable to remove the entire bacterial community from wastewater (109). This may be due to small bacteria or spores passing through the membrane, or to improper membrane installation, allowing bacteria to bypass the filtration barrier and potentially act as a source of contamination. Economically, this tertiary treatment is by far one of the most expensive, with costs ranging from 0.4 to 1 EUR/m³ (102). The high cost can be problematic for lower-income countries that cannot afford such materials and technologies, preventing them from properly using TWW for irrigation.

According to the available data, tertiary treatments and, in particular, the use of membranes are capable of eliminating almost all bacteria including *C. difficile* (16). Nevertheless, more data are needed on Clostridia and their spores in order to be able to conclude definitively.

## CONCLUSIONS

In a context of increasingly pronounced water shortages, the reuse of treated wastewater presents both significant advantages and potential risks. While it offers a sustainable solution for water scarcity and supports agricultural productivity, the presence of pathogenic and/or antibiotic-resistant spore-forming bacteria, such as *Clostridioides difficile* and *Clostridium perfringens*, raises critical health and environmental concerns. These organisms produce highly resistant spores that can survive conventional wastewater treatment processes, and through irrigation may be disseminated in irrigation systems, soils, and crops, potentially representing a risk for human and/or animal health.

While current French and European regulations for wastewater reuse include the enumeration of *C. perfringens*, they do not include *C. difficile* or their characterization. To evaluate the risk associated with these bacteria and wastewater reuse, studies should include an analysis of their toxinotypes and antimicrobial resistance in order to determine the potential risk to human and animal health. Moreover, to ensure wastewater is safe for reuse, treatment processes must be optimized to effectively remove *C. difficile* and *C. perfringens*. Conventional treatment processes, such as sedimentation and activated sludge systems, may achieve partial reduction but are generally insufficient to ensure complete the removal of persistent spore-forming bacteria. Among the promising advanced techniques, membrane filtration stands out as an effective solution. However, its high cost remains a challenge for large-scale application or for lower-income countries. Further research and technological advancements are needed to improve its affordability and integrate it with other treatment methods, such as advanced oxidation processes and biofiltration, to enhance overall efficiency. Optimizing these techniques would contribute to making wastewater safe for agricultural reuse while minimizing health and environmental risks.

There are still significant knowledge gaps regarding the persistence, transmission, and impact of Clostridia in treated wastewater and agricultural environments. Future research is crucially needed in the following areas.

- The development of more efficient pathogen removal technologies in WW: this includes systematic evaluation of advanced treatment barriers specifically against persistent spore-forming bacteria. Controlled pilot-scale studies and quantitative microbial risk assessment frameworks should be used to determine removal efficiencies for *C. difficile* and *C. perfringens*.
- Characterization of clinical and environmental *C. difficile* and *C. perfringens* strains and resistance determinants: integrated surveillance combining culture-based isolation, whole-genome sequencing, and resistance analysis should be implemented to differentiate clinical versus non-clinical lineages and to assess the distribution of antimicrobial resistance genes and toxin genes. Particular attention should be paid to the detection and characterization of mobile genetic elements (e.g., plasmids, transposons) as well as to horizontal gene transfer mechanisms and their potential exchange between clostridial pathogens, gut microbiota, and environmental microbial communities in wastewater systems.
- Monitoring and modeling dissemination in agroecosystems: longitudinal field studies are needed to quantify the persistence and fate of spores in crops, soils, and irrigation systems under treated wastewater reuse scenarios. Coupling environmental monitoring with transport modeling and quantitative microbial risk assessment approaches would enable prediction of exposure pathways and risk under different reuse practices and climatic conditions.

### Recommendations for policymakers and the scientific community

To ensure the safe and sustainable reuse of wastewater, policymakers and researchers must collaborate on the following.

- Establishing and enforcing comprehensive TWW quality regulations using relevant indicators reflecting all categories of pathogens.
- Assessing the current technologies used in wastewater treatment for the control of spore-forming bacteria.
- Encouraging investment in advanced treatment methods, such as membrane filtration and combined disinfection strategies.
- Raising awareness among stakeholders, including farmers, public health officials, and wastewater treatment professionals, to implement best practices for wastewater reuse.

By integrating these measures, TWW reuse can become a safe and viable solution for sustainable agriculture, minimizing health and environmental risks while maximizing resource efficiency, crop productivity, and long-term economic benefits. This approach not only supports water conservation in regions facing scarcity but also contributes to a circular economy and the overall resilience of agricultural systems.
